# Transient isolated brainstem symptoms preceding posterior circulation stroke: a population-based study

**DOI:** 10.1016/S1474-4422(12)70299-5

**Published:** 2013-01

**Authors:** Nicola LM Paul, Michela Simoni, Peter M Rothwell, for the Oxford Vascular Study

**Affiliations:** a Stroke Prevention Research Unit, Nuffield Department of Clinical Neurosciences, John Radcliffe Hospital, Oxford, UK

## Abstract

**Background:**

Transient isolated brainstem symptoms (eg, isolated vertigo, dysarthria, diplopia) are not consistently classified as transient ischaemic attacks (TIAs) and data for prognosis are limited. If some of these transient neurological attacks (TNAs) are due to vertebrobasilar ischaemia, then they should be common during the days and weeks preceding posterior circulation strokes. We aimed to assess the frequency of TNAs before vertebrobasilar ischaemic stroke.

**Methods:**

We studied all potential ischaemic events during the 90 days preceding an ischaemic stroke in patients ascertained within a prospective, population-based incidence study in Oxfordshire, UK (Oxford Vascular Study; 2002–2010) and compared rates of TNA preceding vertebrobasilar stroke versus carotid stroke. We classified the brainstem symptoms isolated vertigo, vertigo with non-focal symptoms, isolated double vision, transient generalised weakness, and binocular visual disturbance as TNAs in the vertebrobasilar territory; atypical amaurosis fugax and limb-shaking as TNAs in the carotid territory; and isolated slurred speech, migraine variants, transient confusion, and hemisensory tingling symptoms as TNAs in uncertain territory.

**Findings:**

Of the 1141 patients with ischaemic stroke, vascular territory was categorisable in 1034 (91%) cases, with 275 vertebrobasilar strokes and 759 carotid strokes. Isolated brainstem TNAs were more frequent before a vertebrobasilar stroke (45 of 275 events) than before a carotid stroke (10 of 759; OR 14·7, 95% CI 7·3–29·5, p<0·0001), particularly during the preceding 2 days (22 of 252 before a vertebrobasilar stroke *vs* two of 751 before a carotid stroke, OR 35·8, 8·4–153·5, p<0·0001). Of all 59 TNAs preceding (median 4 days, IQR 1–30) vertebrobasilar stroke, only five (8%) fulfilled the National Institute of Neurological Disorders and Stroke (NINDS) criteria for TIA. The other 54 cases were isolated vertigo (n=23), non-NINDS binocular visual disturbance (n=9), vertigo with other non-focal symptoms (n=10), isolated slurred speech, hemisensory tingling, or diplopia (n=8), and non-focal events (n=4). Only 10 (22%) of the 45 patients with isolated brainstem TNAs sought medical attention before the stroke and a vascular cause was suspected by their physician in only one of these cases.

**Interpretation:**

In patients with definite vertebrobasilar stroke, preceding transient isolated brainstem symptoms are common, but most symptoms do not satisfy traditional definitions of TIA. More studies of the prognosis of transient isolated brainstem symptoms are required.

**Funding:**

Wellcome Trust, UK Medical Research Council, Dunhill Medical Trust, Stroke Association, National Institute for Health Research (NIHR), Thames Valley Primary Care Research Partnership, and the NIHR Biomedical Research Centre, Oxford.

## Introduction

The diagnosis of transient ischaemic attack (TIA) is often based purely on the description of symptoms, particularly in patients with vertebrobasilar events in whom positive diffusion weighted imaging is less common^1^, ^2^ and inter-observer agreement in diagnosis by experienced neurologists is only moderate.^3^, ^4^ Rapid access to secondary prevention after TIA reduces the risk of early recurrent stroke^5^, ^6^ and is recommended in clinical guidelines.^7^, ^8^, ^9^, ^10^, ^11^, ^12^, ^13^ However, a false-positive diagnosis of TIA can adversely affect patient confidence, employment, lifestyle, and insurance costs and can result in unnecessary preventative treatments. Nevertheless, missing the diagnosis means that the opportunity to prevent a disabling stroke is lost. Indeed, evidence exists that apparently non-TIA transient neurological attacks (TNAs) are associated with an increased risk of stroke, coronary events, and dementia.^14^, ^15^, ^16^

The inconsistency in clinical diagnosis of vertebrobasilar TIA is due mainly to uncertainty about the nature of several transient isolated brainstem symptoms. Most clinical guidelines do not specify which of these symptoms should be deemed as TIA,^7^, ^8^, ^9^, ^10^, ^11^, ^12^, ^13^ although some cite the National Institute of Neurological Disorders and Stroke (NINDS) criteria.^17^, ^18^ The NINDS criteria state that most transient isolated brainstem symptoms, such as vertigo, dysarthria, dizziness or wooziness, focal symptoms suggestive of migraine, confusion, and amnesia, should not be defined as TIAs,^19^ although many clinicians take a more flexible view in routine practice. Previous studies of the generality of TIAs or TNAs have not looked specifically at isolated transient brainstem symptoms and there have been no prospective unselected population-based studies of incidence and prognosis on which to base clinical decisions. Indeed, such studies would be difficult because many (probably most) patients with transient neurological symptoms do not report them to medical attention. However, one useful epidemiological approach, which was helpful in confirming the high risk of stroke after clinically definite TIA,^20^ is to determine the frequency of events during the days and weeks immediately preceding a stroke. This temporal relation can be estimated fairly reliably because most patients do present to medical attention after a stroke,^20^ and it also focuses on the most clinically important subset of TNAs—ie, those that are associated with impending stroke, irrespective of whether the individual sought medical attention after the warning symptoms. We therefore aimed to assess the frequency of isolated brainstem TNAs preceding vertebrobasilar stroke and to compare the frequency with the background rate of such symptoms in patients with stroke in the carotid territory.

## Methods

### Study design and patients

The Oxford Vascular Study (OXVASC) is a prospective, population-based study of all stroke and TIA in 91 105 individuals of all ages registered with 63 primary care physicians in Oxfordshire, UK. We restricted analysis to patients with a first stroke presenting to medical attention from April 1, 2002, to March 31, 2010.

The study methods have been described elsewhere.^21^, ^22^ Briefly, various overlapping methods of hot and cold pursuit were used to achieve near complete ascertainment of all individuals presenting to medical attention with TIA or stroke.^21^, ^22^ These included:

(1) An open-access TIA service available 7 days per week to which participating general practitioners and the local accident and emergency department send all individuals with suspected TIA or minor stroke whom they would not usually admit to hospital, with emergency provision at weekends supplementing a weekday clinic; (2) daily searches of admissions to the stroke unit, general medical, neurology, and other relevant wards; (3) daily searches of the local accident and emergency department and eye hospital attendance register; (4) monthly computerised searches of general practitioner diagnostic coding and hospital discharge codes; (5) monthly searches of all cranial and carotid imaging studies done in local hospitals; and (6) monthly reviews of all death certificates and coroners' reports.

OXVASC was approved by our local ethics committee (Oxfordshire Ethics Committee A: 05/Q1604/70). All patients provided written informed consent and were seen by study physicians as soon as possible after their initial presentation. Relatives provided written assent and clinical details for those unable to provide written consent.

### Procedures

The study senior neurologist (PMR) reviewed all cases and classified them as stroke or other disorder using standard definitions.^21^ Severity of event was assessed with the National Institutes of Health stroke scale (NIHSS)^23^ and clinical features. We classified events as minor stroke if there was a focal neurological deficit lasting more than 24 h and an NIHSS score of 5 or lower^5^ at time of assessment by a study physician. We classified vascular territory (carotid or vertebrobasilar) of stroke on the basis of brain imaging and by use of the NINDS criteria applied to clinical features of the event.^19^ We excluded strokes of uncertain vascular territory. Vascular assessment was done with CT, MRI, or catheter angiography as clinically appropriate.

A detailed history about preceding events was obtained from every patient with a standardised questionnaire.^21^, ^22^ This questionnaire included date of symptom onset, duration and type of symptoms, baseline characteristics, and time of first seeking medical attention. All patients were asked about TIA or TNA symptoms during the previous 90 days. Additionally, we searched hospital notes and family doctors' records for TIA or TNA symptoms in the previous 90 days for all patients. We prospectively classified TNAs using a predefined, diagnostic classification system. We classified isolated vertigo, vertigo with non-focal symptoms, isolated double vision, transient generalised weakness, and binocular visual disturbance as TNAs in the vertebrobasilar territory. Binocular visual disturbance includes patients with partial or complete visual field loss that is not sufficiently clear cut to be a definite TIA; this category would not include a homonymous hemianopia or quadrantinopia but does include patients with lone bilateral blindness, lone bilateral visual blurring, visual scrolling, or other unusual visual perceptions that are not clearly related to migraine or other non-vascular cause. These cases differ from migraine in that so-called positive symptoms (such as fortification spectra or photopsia) will not be prominent. Atypical amaurosis fugax (atypical monocular visual disturbance, such as isolated visual blurring, a sensation of looking through water) and limb-shaking episodes (presumed to be due to low flow in the ipsilateral carotid artery) were classified as TNAs in the carotid territory. Isolated slurred speech, migraine variants, transient confusion, and hemisensory tingling symptoms were classified as TNAs in uncertain territory. All TNAs were further classified according to whether they fulfilled NINDS TIA criteria.^19^

### Statistical analysis

We compared baseline characteristics of patients with stroke in the vertebrobasilar territory (vertebrobasilar stroke) versus those with stroke in the carotid territory (carotid stroke) using the χ^2^ test for categorical variables and *t* test for age. Where significant differences were noted, appropriately stratified analyses were done. We used the χ^2^ test to compare the frequency and type of TNAs reported during the 90 days preceding a vertebrobasilar stroke versus a carotid stroke and to compare the frequency of events preceding stroke with or without symptomatic arterial stenosis. Given that some patients experience multiple TNAs before stroke, to avoid double counting, analysis was based on the first TNA or TIA during the 90 days preceding stroke, rather than the most recent TNA or TIA. We assumed an arbitrary level of 5% significance.

### Role of the funding source

The funding sources had no role in study design, collection, analysis, and interpretation of data, writing of the report, or the decision to submit for publication. The corresponding author had full access to all of the data in the study and had final responsibility for the decision to submit for publication.

## Results

Of 1141 consecutive patients with definite stroke, vascular territory could be identified in 1034 (91%). Of these, 742 (72%) patients had a minor stroke and 292 (28%) had a major stroke. 759 (73%) patients had a stroke in the carotid territory and 275 (27%) in the vertebrobasilar territory (table 1). Patients with carotid stroke were older than those with vertebrobasilar stroke, but other baseline characteristics were similar (table 1).

**Table 1:** Baseline characteristics, vascular risk factors, and premorbid medication in patients with definite ischaemic stroke, by territory

	**Carotid stroke (n=759)**	**Vertebrobasilar stroke (n=275)**	**p value**
Mean age (years)	75·9 (11·8)	73·3 (13·1)	0·002
Male sex	372 (49%)	133 (48%)	0·88
Hypertension	450 (59 %)	173 (63%)	0·31
Diabetes	85 (11 %)	35 (13%)	0·51
Angina or myocardial infarction	145 (19%)	64 (23%)	0·16
Peripheral vascular disease	59 (8%)	28 (10%)	0·25
Previously diagnosed atrial fibrillation	156 (21%)	50 (18%)	0·43
Current smoker	107 (14%)	34 (12%)	0·54
Previous TIA	65 (9%)	20 (7%)	0·61
Previous stroke	126 (17%)	33 (12%)	0·08
Previous antiplatelet treatment	336 (44%)	109 (40%)	0·20
Previous statin treatment	168 (22%)	61 (22%)	1·0
Previous antihypertensive treatment	443 (58%)	171 (62%)	0·28

Data are number of patients (%) or mean (SD). TIA=transient ischaemic attack.

Events (TIAs and other TNAs) were more common in the 90 days preceding a vertebrobasilar stroke than before carotid stroke (in 59 of 275 patients before a vertebrobasilar stroke *vs* 65 of 759 patients before a carotid stroke; OR 2·92, 95% CI 1·99–4·28, p<0·0001). Of 275 patients with vertebrobasilar stroke, 59 (22%, 95% CI 16·6–26·4) had at least one event within the preceding 90 days (median 4 days, IQR 1–30) of which only five (8%) had events that fulfilled the NINDS criteria for TIA (figure). In the remaining 54 patients, 45 had isolated brainstem TNAs (23 patients with isolated vertigo, nine with non-NINDS binocular visual disturbance, ten with vertigo with other non-NINDS symptoms, two with isolated diplopia, and one with transient generalised weakness), and nine had TNAs of uncertain territory (four patients with isolated slurred speech, two with isolated hemisensory symptoms, two with transient confusion, and one with a migraine variant). In seven (13%) of these 54 patients, multiple TNAs occurred, with isolated vertigo being the most common (four of seven). In all four cases, the vertigo occurred suddenly and without provocation by movement.

**Figure:**
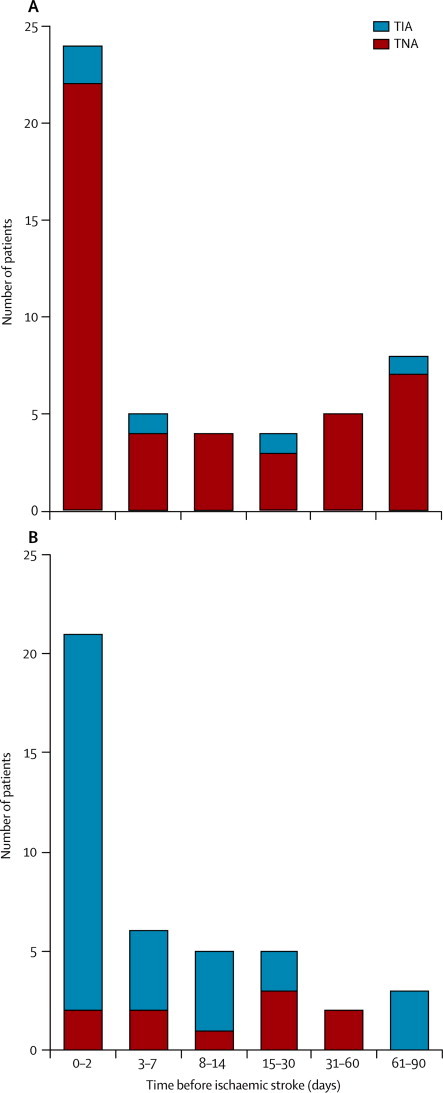
Occurrence of vertebrobasilar TNA or TIA in the 90 days before vertebrobasilar stroke (A) versus occurrence of carotid TNA or TIA in the 90 days before carotid stroke (B) TIA=transient ischaemic attack. TNA=transient neurological attack.

The temporal relation between isolated vertebrobasilar TNA and vertebrobasilar stroke was very similar to that of definite carotid TIA and carotid stroke (figure), both being concentrated during the 2 days preceding the stroke. The rate of definite carotid TIA in the 2 days preceding carotid stroke was about 60 times greater than that during the preceding 3–90 days (9·5 events per day, 19 TIAs in 0–2 days, *vs* 0·15 events per day, 13 TIAs in 3–90 days; relative rate 64·3, 95% CI 31·76–130·21). This relative rate was similar for isolated brainstem TNAs preceding vertebrobasilar stroke (11·0 events per day, 22 TNAs in 0–2 days *vs* 0·26 events per day, 23 TNAs in 3–90 days).

Isolated TNAs in the vertebrobasilar territory were about 15 times more likely before a vertebrobasilar stroke than before a carotid stroke (table 2), slightly more so when cardioembolic strokes were excluded (41 of 219 events before vertebrobasilar stroke *vs* eight of 558 events before carotid stroke, OR 15·8, 95% CI 17·0–37·3, p<0·0001), but strikingly more so for events within the 2 days immediately preceding the stroke (OR 35·8, 8·4–153·5, p<0·0001; table 2). The difference in proportion remained high for events occurring 8–90 days preceding the stroke (OR 9·3, 3·7–23·6, p<0·0001; table 2).

**Table 2:** TNA or TIA in the 90 days preceding stroke by territory

		**Territory of stroke**		**OR (95%CI)**	**p value**
		Vertebrobasilar	Carotid		
TNA in vertebrobasilar territory*		45/275 (16%)	10/759 (1%)	14·7 (7·3–29·5)†	<0·0001
	0–2 days	22/252 (9%)	2/751 (<1%)	35·8 (8·4–153·5)	<0·0001
	3–7 days	4/256 (2%)	2/753 (<1%)	6·0 (1·1–32·7)	0·04
	8–90 days	19/275 (7%)	6/759 (1%)	9·3 (3·7–23·6)	<0·0001
TNA in uncertain territory‡		9/225 (4%)	17/717 (2%)	1·7 (0·8–3·9)	0·20
Definite TIA		5/221 (3%)	32/732 (4%)	0·5 (0·2–1·3)	0·16

TNA=transient neurological attack. TIA=transient ischaemic attack. Data are n/N (%) unless otherwise stated.

* Isolated vertigo, vertigo plus other symptoms of any type, isolated double vision, transient generalised weakness, and binocular visual disturbance. Days indicate the time of the transient event before the stroke event.

† This association was independent of age: OR 12·8 (95% CI 3·6–45·9) for ages <65 years; OR 14·7 (7·3–29·5) for ages 65–74 years; and OR 11·0 (4·6–26·4) for ages ≥75 years.

‡ Isolated slurred speech, migraine variant, transient confusion, and isolated hemisensory tingling. Denominators are based on first TNA or TIA during the 90 days preceding stroke; therefore patients with TNAs or TIAs during the initial period were censored.

TNAs thought to be of carotid territory origin, such as atypical amaurosis fugax and limb shaking episodes, occurred only before definite carotid stroke (six of 759 patients, three with atypical amaurosis fugax and three with limb-shaking). Of the 759 patients with definite carotid territory stroke, 65 (9%) had at least one TNA or TIA within the preceding 90 days (median 7·0 days, IQR 1–19·5) of which 32 (49%) fulfilled the NINDS criteria for TIA (figure, table 2).

Only ten (22%) of the 45 patients with isolated brainstem TNAs sought medical attention before the stroke and a vascular cause was suspected by their physician in one of these cases. In the remainder of cases, the initial diagnosis was documented as postural hypotension (three cases), peripheral vestibular disturbance (two cases), sepsis (two cases), migraine (one case), and cranial nerve palsy (one case). Vascular risk factors were more common in those with isolated vertigo, compared with other vertebrobasilar TNAs (table 3).

**Table 3:** Vascular risk factors and duration of isolated vertigo versus other vertebrobasilar TNAs preceding vertebrobasilar stroke

		**Isolated vertigo (n=23)**	**All other vertebrobasilar TNA (n=22)**
Age ≥60 years		17 (74%)	15 (68%)
Any vascular risk factors (diabetes mellitus, hypertension, smoking, or atrial fibrillation)		19 (83%)	11 (50%)
Previous vascular events (myocardial infarction, peripheral vascular disease, TIA, or stroke)		4 (17%)	9 (41%)
Any vascular medication*		12 (52%)	17 (77%)
Duration of symptoms			
	<1 h	11 (48%)	15 (68%)
	≥1 h	12 (52%)	7 (32%)

Data are number of patients (%) unless otherwise stated. TNA=transient neurological attack. TIA=transient ischaemic attack.

* Any antiplatelet, statin, or antihypertensive.

In patients with stroke in whom 50% or higher symptomatic stenosis was documented, TNAs were also more frequent preceding vertebrobasilar stroke than preceding carotid stroke (table 4). This excess remained when patients with definite TIA and 50% or higher symptomatic stenosis were added to the analysis (table 4). Among the 16 patients with vertebrobasilar TIA and 50% or higher symptomatic vertebrobasilar stenosis, preceding TNAs were more commonly binocular visual disturbance (n=7) than isolated vertigo (n=2).

**Table 4:** Events in the 90 days preceding ischaemic stroke or TIA with ≥50% symptomatic stenosis

	**Strokes with ≥50% symptomatic stenosis**				**Strokes and TIAs with ≥50% symptomatic stenosis**			
	Vertebrobasilar stenosis (n=39)	Carotid stenosis (n=45)	OR (95% CI)	p value	Vertebrobasilar stenosis (n=72)	Carotid stenosis (n=95)	OR (95% CI)	p value
NINDS TIA	1 (3%)	4 (9%)	0·27 (0·01–2·8)	0·37	5 (7%)	17 (18%)	0·3 (0·1–1·1)	0·04
TNA	11 (28%)	3 (7%)	5·50 (1·3–27·6)	0·02	27 (38%)	12 (13%)	4·2 (1·8–9·7)	0·002
No TIA or TNA	27 (69%)	38 (84%)	0·41 (0·1–1·3)	0·12	40 (56%)	66 (69%)	0·5 (0·3–1·1)	0·08

Data are n (%) unless otherwise stated. TNA=transient neurological attack. TIA=transient ischaemic attack. OR=odds ratio. NINDS=National Institute of Neurological and Disorders and Stroke. Vascular evaluation was done using CT, MRI, or catheter angiography as clinically appropriate. Stenosis was defined as 50% or greater symptomatic stenosis. Derived from a total of 873 strokes with vascular imaging and 83 TIAs with 50% or greater symptomatic stenosis.

## Discussion

Our study shows that there is a temporal relation between transient isolated brainstem symptoms and vertebrobasilar stroke (panel). Such events occurred in the days and weeks immediately preceding 16% of vertebrobasilar strokes. Of all events occurring during the 90 days preceding a vertebrobasilar stroke, fewer than 10% satisfied the NINDS definition of TIA. We also noted that most patients with such symptoms failed to present to medical attention at the time but presented only after a subsequent stroke, potentially leading to major bias in determining risk prospectively even in a population-based study of TNAs.

Panel **Research in context**

**Systematic review**

We searched Medline (1950–2011) and Embase (1988–2011) and also hand-searched relevant journals and the reference lists of included papers using the search terms “transient ischaemic attack”, “transient neurological attack (TNA)”, “stroke”, “vertebrobasilar”, and “stroke symptoms”. Searches were restricted to human studies. All types of study with at least three patients were included. Several studies^14^, ^15^, ^16^, ^24^, ^25^, ^26^, ^27^, ^31^, ^32^, ^33^, ^34^, ^35^, ^36^, ^37^, ^38^ have shown an association between TNAs and stroke, particularly isolated vertigo, but such TNAs do not satisfy traditional definitions of TIA.

**Interpretation**

We studied all potential ischaemic events during the 90 days preceding an ischaemic stroke in patients ascertained within a prospective, population-based incidence study in Oxfordshire, UK (the Oxford Vascular Study). Isolated brainstem TNAs were more frequent before a vertebrobasilar stroke than before a carotid stroke, particularly during the preceding 2 days. Our study shows a close temporal relation between vertebrobasilar stroke and preceding isolated brainstem transient neurological attacks that do not satisfy the NINDS definition of TIA. More studies of the prognosis of the different transient isolated brainstem symptoms are needed.

Results from large cohort studies have shown that 12–18% of people without a previous diagnosis of stroke reported TIA-like symptoms.^24^, ^25^, ^26^, ^27^ However, none of these studies determined the risk of stroke during the subsequent days and weeks and there are very few prospectively collected data of the prognosis after TNA. Prospective studies can underestimate stroke risk by missing those who do not seek medical attention at the time of the TNA. Questionnaire-based surveys of patients looking at the frequency of previous TNAs have various recall biases and exclude those who had a major or fatal stroke after the TNA and are therefore unable to respond.^24^, ^25^, ^26^, ^27^ Given these difficulties, large prospective population-based studies with complete ascertainment are probably impractical. However, it is possible to identify and study the most important subset of TNAs—ie, those that are associated with a subsequent stroke and which should therefore not be missed or misdiagnosed. Since half of all recurrent strokes that occur in the 5 years after a definite TIA occur in the first 90 days,^28^ with a similar front-loaded risk after vertebrobasilar TIA,^29^, ^30^ if we assume a similar time course for atypical TIAs (or TNAs) then useful information on the nature of high-risk TNAs can be gleaned from studying those events that occur in the 90 days preceding a definite stroke.

Isolated vertigo was the most common transient symptom preceding vertebrobasilar stroke in our study. Results from the Atherosclerosis Risk in Communities Study (ARIC) study^24^ showed that dizziness was the most frequently reported atypical symptom (36%) in the underlying population, although only 1·2% were classified as having a TIA or stroke.^24^ In elderly people, the prevalence of vertigo in the year before stroke has been reported to be 15%.^31^ Results from a prospective study of patients admitted to hospital with isolated vertigo showed a three-fold increase in stroke risk compared with controls up to 4 years after hospital discharge.^32^ Results from a retrospective study of patients with vertigo identified from MRI requests made after emergency department attendance showed 9·2% had acute stroke. Gait instability and subtle neurological examination findings were associated with increased stroke risk.^33^ Studies using Doppler sonography, magnetic resonance angiography, or catheter angiography have shown that isolated vertigo can be the only manifestation of vertebrobasilar ischaemia.^33^, ^34^, ^35^, ^36^ In a study of 29 patients admitted to hospital with possible vertebrobasilar TIA, isolated vertigo was present in 21%.^34^ In our study, the close temporal relation between vertigo and vertebrobasilar stroke suggests that these TNAs are often secondary to vertebrobasilar ischaemia.

Our findings have implications for public education to improve recognition and awareness of the significance of symptoms of TIA. In our study, of those patients who did present to medical attention after the initial event, most were not managed as having TIAs, suggesting that broader diagnostic criteria are needed. Although we do not know the rate of TNAs in the population, in patients with isolated vertigo plus vascular risk factors, referral for specialist opinion would appear to be reasonable.

The main strengths of our study were the population-based design, the rigorous case-ascertainment, and the prospective definition and classification of the various types of TNA. However, our study has limitations. First, the prevalence of TNAs before stroke events might be inaccurate if patients failed to report them during direct questioning or if recall bias increased recollection and reporting of TNAs. However, the very rapid fall in rate of preceding TNAs over the few days before the stroke is not what would be expected on the basis of recall bias—people would recall events for the preceding few weeks and not just the preceding few hours. Most importantly, in previous studies of definite TIA before stroke, this same time course of preceding TIA was noted^20^ and was subsequently confirmed in prospective studies.^21^ We might also have missed TNAs in patients who were unable to give a clear history after stroke because of aphasia or reduced consciousness. Second, although our attribution of the probable vascular territory of TNAs was predefined, it might not be accurate in all cases. Finally, we do not have data of the total number of patients with TNAs in our population who sought medical attention but were not considered to have TNAs and were not referred to secondary care during the period of the study. Without these data on the denominator we cannot identify the absolute risk of vertebrobasilar stroke after an isolated brainstem TNA reported to medical attention. Moreover, we do not, of course, know how many patients had such symptoms and did not seek medical attention. Furthermore, a previous study of patients with TNAs who were referred to hospital suggested that family doctors tend to refer those with vascular risk factors, potentially leading to a referral bias.^37^ Nevertheless, in those studies that have attempted to determine the incidence of TNAs that are reported to medical attention, the numbers of events have been similar to the number of definite TIAs ascertained over the same period,^38^ suggesting that the number of denominator events (ie, the total number of TNAs in the population) might not be so large as to substantially dilute the risk of stroke.

In conclusion, our study shows a close temporal relation between vertebrobasilar stroke and preceding isolated brainstem TNAs which do not satisfy the NINDS definition of TIA. A high index of suspicion is particularly important in view of the short-time window available for prevention of stroke. However, because of the adverse effects of a false-positive diagnosis of TIA, more studies of the prognosis of the different transient isolated brainstem syndromes are needed.
